# Cognitive State following Stroke: The Predominant Role of Preexisting White Matter Lesions

**DOI:** 10.1371/journal.pone.0105461

**Published:** 2014-08-25

**Authors:** Efrat Kliper, Einor Ben Assayag, Ricardo Tarrasch, Moran Artzi, Amos D. Korczyn, Shani Shenhar-Tsarfaty, Orna Aizenstein, Hen Hallevi, Anat Mike, Ludmila Shopin, Natan M. Bornstein, Dafna Ben Bashat

**Affiliations:** 1 Functional Brain Center, The Wohl Institute for Advanced Imaging, Tel Aviv Sourasky Medical Center, Tel Aviv, Israel; 2 Sackler Faculty of Medicine, Tel Aviv University, Tel Aviv, Israel; 3 Neurology Department, Tel Aviv Sourasky Medical Center, Tel Aviv, Israel; 4 Jaime and Joan Constantiner School of Education, Tel Aviv University, Tel Aviv, Israel; 5 Sagol School of Neuroscience, Tel Aviv University, Tel Aviv, Israel; University of Texas at Dallas, United States of America

## Abstract

**Background and purpose:**

Stroke is a major cause of cognitive impairment and dementia in adults, however the role of the ischemic lesions themselves, on top of other risk factors known in the elderly, remains controversial. This study used structural equation modeling to determine the respective impact of the new ischemic lesions' volume, preexisting white matter lesions and white matter integrity on post stroke cognitive state.

**Methods:**

Consecutive first ever mild to moderate stroke or transient ischemic attack patients recruited into the ongoing prospective TABASCO study underwent magnetic resonance imaging scans within seven days of stroke onset and were cognitively assessed one year after the event using a computerized neuropsychological battery. The volumes of both ischemic lesions and preexisting white matter lesions and the integrity of the normal appearing white matter tissue were measured and their contribution to cognitive state was assessed using structural equation modeling path analysis taking into account demographic parameters. Two models were hypothesized, differing by the role of ischemic lesions' volume.

**Results:**

Structural equation modeling analysis of 142 patients confirmed the predominant role of white matter lesion volume (standardized path coefficient *β = −0.231*) and normal appearing white matter integrity (*β = −0.176*) on the global cognitive score, while ischemic lesions' volume showed no such effect (*β = 0.038*). The model excluding the ischemic lesion presented better fit to the data (comparative fit index 0.9 versus 0.092).

**Conclusions:**

Mild to moderate stroke patients with preexisting white matter lesions are more vulnerable to cognitive impairment regardless of their new ischemic lesions. Thus, these patients can serve as a target group for studies on cognitive rehabilitation and neuro-protective therapies which may, in turn, slow their cognitive deterioration.

## Background

Cognitive impairment or dementia affects approximately 30% of stroke patients [1], [2], [3], [4], , but the role of the ischemic lesions' volume (ILV) on top of other risk factors known in the elderly, remains open to debate. Several studies showed a direct relationship between ILV and patient's cognitive state [5], [7], [8], [9], while others rejected this finding [10], [11]. White matter lesions (WML), on the other hand, are known as major contributors to cognitive impairment and dementia amongst stroke survivors and the elderly in general [12], [13], [14], [15]. WML may indicate small vessel vascular brain disease [16], demyelination or inflammatory processes [17]. WML are a common finding in the elderly [12], [18] and are found in up to 44% of patients with stroke or transient ischemic attack (TIA) and in 50–75% of patients with vascular dementia [14], [19]. The effect of WML load on cognitive function was shown regardless of the cognitive domain assessed [20].

WML, which appear as hyperintense signal on T2-weighted images (WI) on magnetic resonance imaging (MRI) may affect the integration of information from large-scale networks [21], thus result in cognitive dysfunction [18], [22], [23]. Diffusion tensor imaging (DTI) enables indirect assessment of white matter (WM) microstructural changes, extending beyond the areas that appear hyperintense on MRI [24], thus allowing quantitative assessment of tissue integrity, and may provide additional information on the relation between subtle changes in WM integrity and cognition. Studies have shown that the degree of WM integrity as evaluated using DTI can improve the prediction of subjects' cognitive state [23], [25]. In stroke, DTI data demonstrated that the degree of damage can add to the prediction of clinical outcome [26], [27].

Structural equation modeling (SEM), and specifically path analysis, provide an effective and direct way of modeling mediation, indirect effects, and more complex relationships among variables [28]. SEM has been used extensively in the social and behavioral sciences and is recently used more widely in cognitive aging research [29], [30], [31]. In this study SEM was used to investigate the relative contribution of new ILV on top of preexisting WML and WM integrity parameters to cognitive state one year after the acute stroke. We hypothesized that first ever stroke or TIA patients who already have extensive WM damage are more likely to demonstrate cognitive impairment regardless of the volume of their ischemic lesions.

## Methods

### Ethics statement

This study was approved by the Tel Aviv Sourasky Medical Center ethics committee. Written informed consent was obtained from all participants.

### Study population

This study was based on participants from the Tel Aviv Brain Acute Stroke Cohort (TABASCO) study [32], an ongoing prospective study of first-ever mild to moderate ischemic stroke or TIA patients aiming to identify predictors for the development of post-stroke cognitive impairment. Patients were excluded if they were younger than 50 years old; suffered from hemorrhagic stroke or stroke resulting from trauma or invasive procedures; had severe aphasia, had pre-stroke history consistent with dementia; if they were unlikely to be discharged home from hospital following the qualifying stroke; or had a severe physical disability making follow-up unlikely.

Between April 2008 and September 2011 a total of 433 consecutive patients were admitted to Tel Aviv Medical Center with a final diagnosis of TIA or mild to moderate stroke (National Institutes of Health Stroke Scale (NIHSS)<17) and met the study inclusion/exclusion criteria. For each patient, demographic data were collected (age, years of education, etc.), medical history and comorbidities as described in Ben Assayag et al. [32]. Neurological assessment included verification of stroke etiology and NIHSS. For the present study 291 patients were excluded: 104 patients who did not have MRI data, 13 patients died, 99 were not available for one year cognitive follow-up and in 75 patients the imaging data were of poor quality due to patient movements or one or more of the sequences essential for the data analysis algorithms was missing. The patients excluded from the present analyses were slightly older (68.7+10.4 vs. 66+9.5, p = 0.01) and less educated (12.7+4 vs. 13.7+3.7, p = 0.02), but with similar neurological deficit at admission (2.7+2.9 vs. 2.6+3, p = 0.675). In total, 142 patients were included in the present analysis, all of whom underwent one year cognitive assessment.

### Vascular risk factors

Vascular risk factors were assessed according to the Framingham Stroke Risk Profile (FSRP) score [33]. FSRP is based on the following risk factors: age, systolic blood pressure, antihypertensive medication, diabetes, cigarette smoking, history of cardiovascular disease, atrial fibrillation, and left ventricular hypertrophy as determined by ECG. As ECG data were not available, and since FSRP is purely additive in nature, the left ventricular hypertrophy was removed from the present analysis. Vascular risk factor data was recorded as described in Ben Assayag et al. [32] and for each patient the FSRP score was calculated.

### MRI acquisition and processing

All images were acquired within 7 days of stroke onset on a 3T GE scanner (GE Signa EXCITE, Milwaukee, WI, USA) using an 8-channel head coil. The protocol consisted of the following pulse sequences: Axial fast Spin-Echo (FSE) T2- weighted images (WI) (Time to repeat (TR)/Time to echo (TE) = 13000/110milliseconds (msec.), matrix = 512×256), fluid-attenuated inversion recovery (FLAIR) (TR/TE/inversion time = 10000/110/2500 msec, matrix = 256×256), T2* Gradient Echo (GE) images (TR/TE = 325/15 msec, matrix = 256×192). All sequences with field of view (FOV) = 240 and slice thickness of 4 mm with no gap. High resolution 3D T1-WI spoiled GE sequences (SPGR) were obtained at axial orientation (TR/TE = 8.976/3.488 msec, FOV = 256, matrix = 256×256 and slice thickness of 1 mm) and coronal orientation (TR/TE = 6.35/1.8 msec, FOV = 250, matrix = 256×256 and slice thickness of 2 mm). Diffusion weighted imaging (DWI) (TR/TE = 6000/72.4 msec, Matrix = 128×128, b values = 0, 1000 sec/mm^2^) and DTI (TR/TE = 12000/89 msec, matrix = 128×128, b values = 0, 1000 sec/mm^2^, and 15 gradients directions) were obtained using echo planar imaging (EPI) sequence, both with field of view (FOV) = 240 and slice thickness of 4 mm with no gap. All axial slices were prescribed on the same orientation, covering the whole brain, aligned along the fourth ventricle-orbitofrontal orientation.

### Ischemic infarct identification

Presence of acute ischemic infarcts was assessed by a senior neuro-radiologist (O.A.), based on the DWI images. Ischemic lesions were defined as cortical (non lacunar), sub-cortical or subtentorial infarcts according to their anatomical locations based on structural MRI images. Cortical infarcts were defined as any infarct that includes the cortex, subtentorial infarcts were defined as cerebellar or brainstem infarction.

### DTI analysis

Calculation of the DTI maps was performed using FMRIB Diffusion Toolbox, part of FMRIB Software Library (FSL, http://www.fmrib.ox.ac.uk/fsl/), and included eddy current and motion correction. Four different maps: fractional anisotropy (FA), mean diffusivity (MD), axial and radial diffusivities (Da and Dr) were calculated.

### Tissue segmentation

The identification and quantification of the ischemic lesions, WML and normal appearing white matter (NAWM) was performed using a multi-modal view with an in-house semi-automatic method. A detailed description of the method can be found in Artzi et al. [34]. Briefly, this methodology uses FLAIR images, on which WML are best visualized as hyperintense regions, and incorporate data obtained from GE T_2_^*^ WI, DWI data and DTI maps. Preprocessing included registration of GRE-T2* WI, DWI and diffusivity maps to the FLAIR images using SPM5 (www.fil.ion.ucl.ac.uk/spm/software/spm5) and FSL linear image registration tool. Skull stripping was performed on the realigned GRE-T2* images using FMRIB's brain extraction tool. The output GRE-T2* brain mask was used to mask all other images and represented the total intracranial volume (ICV). Non-uniformity correction using N3 MINC B0 tool (part of Freesurfer, http//www.nitrc.org/projects/freesurfer) was applied on the FLAIR images. Unsupervised clustering into two clusters was performed on the preprocessed FLAIR images, separating (1) NAWM + normal appearing gray matter (NAGM) and (2) cerebrospinal fluid and hyperintense FLAIR areas. The mean and standard deviation (SD) values of the NAWM+NAGM were calculated. Threshold based segmentation was performed on the FLAIR images to classify voxels into WML (2 SD above the mean value of the NAWM+NAGM) and ischemic lesions (2 SD above the mean value of the NAWM+NAGM in addition to hyperintensity on DWI). In order to identify the NAWM area and to minimize partial volume effect, a threshold was applied to the FA maps to include only voxels with FA value >0.2, and this was used to mask the obtained NAWM+NAGM area. Additionally, all segmentation results were visually inspected and manually corrected.

**Figure 1:**
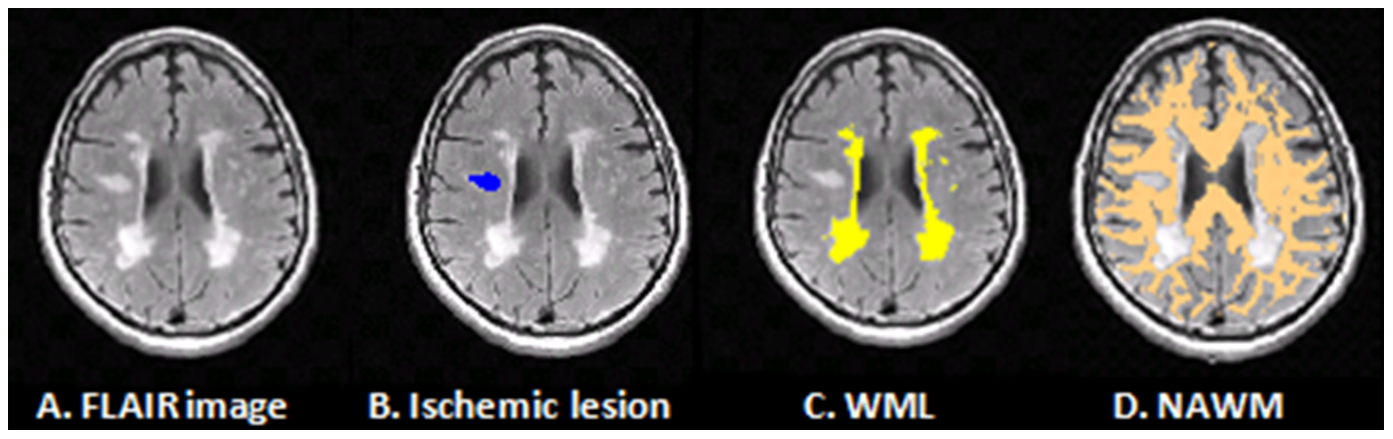
Tissue segmentation example. Subtext: A 60 year old male, scanned two days following stroke onset (A) FLAIR, fluid-attenuated inversion recovery image; (B) Ischemic lesion (blue); (C) WML, white matter lesions (yellow); (D) NAWM, normal appearing white matter (copper).

Volumes (in mm^3^) of both ischemic lesions and WML were calculated across the whole brain (see Figure 1 for segmentation output example). Relative volumes of ischemic lesions and WML were calculated by dividing the total volume of each tissue cluster by the ICV. Mean FA, MD, Dr and Da values were calculated for WML and NAWM tissue clusters.

### Cognitive assessments

All patients performed NeuroTrax™ computerized cognitive testing during hospitalization and one year after the event. NeuroTrax™ (NeuroTrax Corp., Bellaire, TX) is a computerized battery of neuropsychological tests that is used for reliable detection of cognitive state in cognitively healthy, mild cognitive impairment and mild dementia subjects [35]. NeuroTrax tests, previously known as MindStreams, provide an overall measure of cognitive function (global cognitive score) as well as evaluation of specific cognitive domains (memory, executive function, verbal function, visual spatial and attention). Each domain score, including the global cognitive score is normalized to fit a standardized scale (mean, 100; SD, 15) in an age- specific fashion. The mean score of 100 points was calculated from a normative sample of approximately 1,600 cognitively healthy individuals as determined by expert diagnosis in research studies with the computerized battery [35].

The current analysis was performed only on the one year cognitive assessment, which is assumed to be less affected by non-specific changes such as stress and insomnia associated with hospital admission, thus more accurately reflects cognitive state.

### Statistical analysis

Statistical analysis was conducted with SPSS for Windows software (version 19.0). Pearson (for interval scaled variables) or Spearman (for ordinal variables) correlations were used to assess relationships between ischemic lesions, WML and NAWM volumes, diffusivity parameters and the different cognitive domain scores one year after the event. Since the volumes of both ischemic lesions and WML were skewed, a logarithmic transformation was applied aiming to normalize the distributions. In order to reduce the probability of false positives due to multiple comparisons, a strict statistical threshold of *p*<0.005 was used.

Analysis of variance (ANOVA) was used to compare the global cognitive score at one year between four different groups of patients categorized by ischemic lesions' location (absence of new infarct, cortical, sub-cortical or subtentorial infarcts).

To test hypothesized models for the prediction of cognitive state in post stroke patients, SEM was employed using the STATISTICA software package (version 9.0). SEM assesses the covariance structure of the variables in question in order to confirm whether the observed data are consistent with the hypothesized model. SEM requires at least 10–20 cases for each path in the model. Therefore, based on our sample size, we have limited our models to seven paths. The parameters that were chosen for assessing a good model fit were: non-significant *p*-value for chi-squared goodness of fit statistic (>0.05), comparative fit index (CFI)>0.95, goodness of fit index GFI>0.95, adjusted GFI (AGFI)>0.95 and root mean square error of approximation (RMSEA)<0.08.

**Figure 2:**
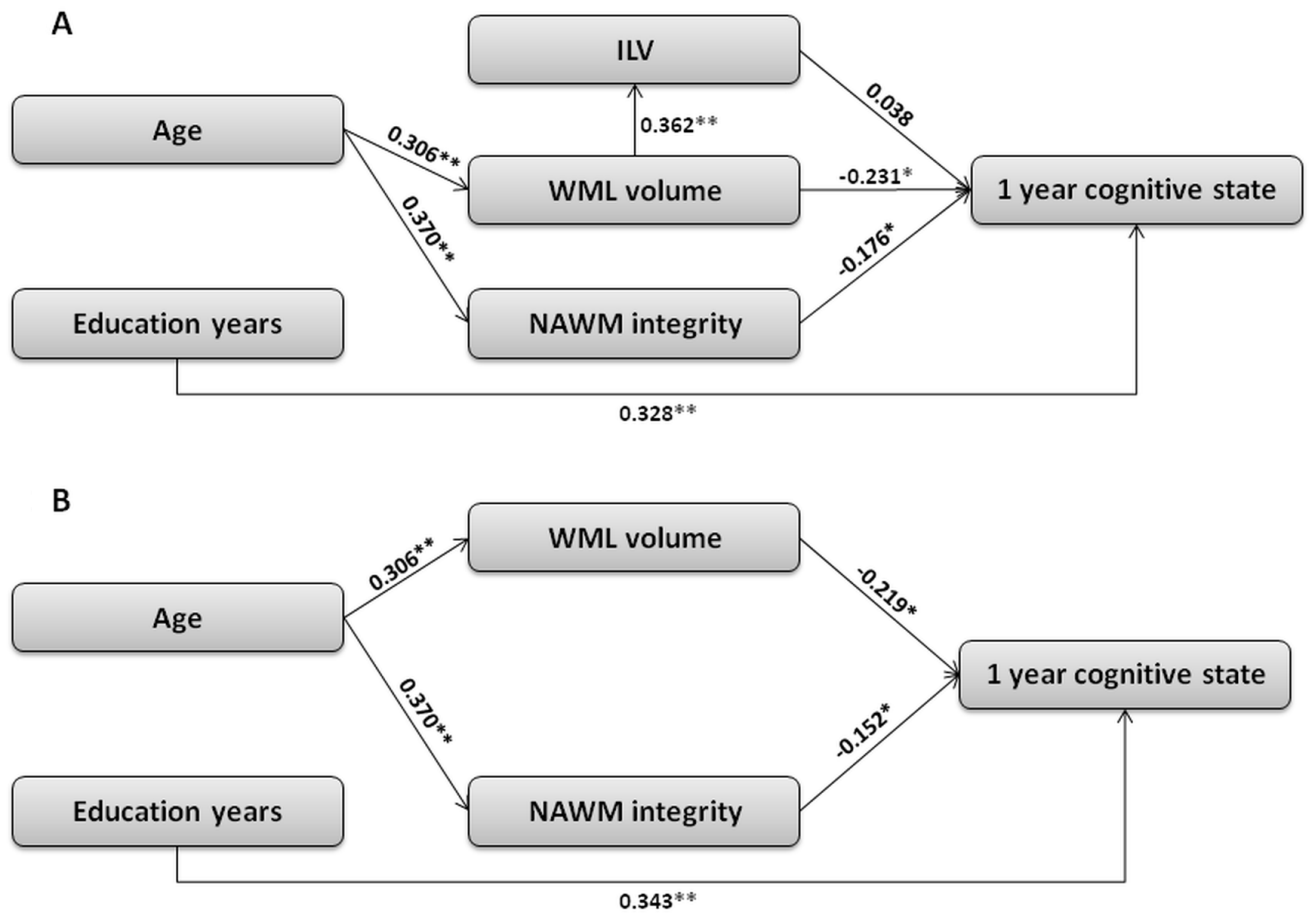
Structural equation models for the prediction of cognitive state after one year. Subtext: The numbers on the arcs represent the contribution of each parameter to its neighbor. * *p*<0.05 **p<0.001. Abbreviations: ILV, ischemic lesions' volume; WML, white matter lesions; NAWM, normal appearing white matter.

Two models were tested (Figure 2) regarding the role of ILV on top of WML volume and the integrity of NAWM in cognitive state as measured in a one year follow-up. The first model (Figure 2A) is based on existing evidence from the literature in which age affects WML volume and NAWM integrity [29], [36], [37], [38], which in turn affect cognitive state [15], [22], [23], [39], [40], [41], and on the well established influence of education level on cognitive state [42], [43], [44]. The model also accounts for the association of WML with an increased risk for stroke [12], [41] and examines the direct effect of ILV on one year global cognitive score. The second model (Figure 2B) is similar to the first but does not take into account the effect of ILV on cognition.

Since all NAWM diffusivity parameters were highly correlated, a principal components factor analysis was performed on the diffusion tensor's eigenvalues λ_1_, λ_2_ and λ_3_ to create a single parameter named NAWM integrity. Similarly, to avoid multicollinearity, the global cognitive score was chosen to represent the cognitive outcome rather than each of the cognitive scores separately.

In order to assess the ability of the baseline parameters to predict the global cognitive state after one year, two sets of multiple linear regressions were carried out. First, unadjusted regression (correlation) coefficients were calculated for demographic and clinical variables that were previously shown to impact the global cognitive score after one year: age, gender, years of education, admission NIHSS, ICV, WML volume, infarct volume, FSRP score, NAWM integrity and WML integrity. Following identification in unadjusted regression, only significant variables (*p*<0.05) were entered concomitantly into the multiple linear regression analyses. The dependent variable was the patient's global cognitive state at one year.

To further test our hypothesis regarding the effect of the ILV on cognitive outcome, analysis of covariance (ANCOVA) was performed with one year cognitive state as a dependent variable, presence of ischemic lesions on DWI (with and without ischemic lesions) as an independent variable and WML volume as covariate.

## Results

### Patients' characteristics

Of the 142 patients, 101 (∼71%) were clinically diagnosed with ischemic stroke and 41 (∼29%) with TIA. Mean age was 65.9 (±9.5) years and 53.1% were males. Other demographic, clinical and cognitive characteristics of the patients are summarized in Table 1.

**Table 1:** Study population demographic and clinical measures, cognitive scores and brain MRI characteristics (data shown as mean ± SD or as number of cases and percentage n (%)).

Demographic and clinical measures	(N = 142)
Age, years	65.9±9.5
Education, years	13.6±3.7
Admission NIHSS	2.5±3.0
Systolic blood pressure mmHg	140±20
Gender, male, n (%)	76 (53.1%)
Risk factors	
Hypertension, n (%)	82 (57.3%)
Diabetes mellitus, n (%)	29 (20.3%)
Dyslipidemia, n (%)	81 (56.6%)
History of heart disease, n (%)	22 (15.5%)
Atrial fibrillation n (%)	13 (9.2%)
Smoking, n (%)	26 (18.1%)
Body mass index >25, n (%)	95 (66.3%)
Infarct Location	
No infarct	50 (36%)
Cortical infarct	30 (21%)
Sub-cortical infarct	48 (33%)
Subtentorial infarct	14 (10%)
**Cognitive score (1 year follow-up)**	
Global cognitive score	96.7±12.4
Memory score	97.9±15.7
Executive function score	98.1±12.0
Verbal function score	90.2±22.8
Visual spatial score	98.5±18.6
Attention score	98.8±13.2
**Brain MRI characteristics**	
Ischemic lesions volume, mm^3^	4949±13741
ICV, mm^3^	1348527±141014
Relative ischemic lesions volume, % of ICV	0.366±1.04
WML volume, mm^3^	18203±17727
Relative WML, % of ICV	1.3±1.3
WML FA values, arbitrary units	0.29±0.05
WML MD values, mm^2^/sec	0.0012±0.0001
WML eigenvalue1 (Da), mm^2^/sec	0.0016±0.00018
WML Dr values, mm^2^/sec	0.0010±0.00017
WML eigenvalue2, mm^2^/sec	0.0011±0.00017
WML eigenvalue3, mm^2^/sec	0.00097±0.00017
NAWM FA values, arbitrary units	0.40±0.01
NAWM MD values, mm^2^/sec	0.0008±0.00003
NAWM eigenvalue1 (Da) values, mm^2^/sec	0.0012±0.00005
NAWM Dr values, mm^2^/sec	0.0006±0.00004
NAWM eigenvalue2, mm^2^/sec	0.00078±0.00004
NAWM eigenvalue3, mm^2^/sec	0.00055±0.00004

Abbreviations: NIHSS, National Institutes of Health Stroke Scale; ICV, intracranial volume; WML, white matter lesions; FA, fractional anisotropy; MD, mean diffusivity; Dr, radial diffusivity; Da, axial diffusivity; NAWM, normal-appearing white matter.

### Tissue parameters

Based on the DWI data, acute ischemic lesions were detected in 92 out of 142 patients by a senior neuro-radiologist. The semi automatic method identified 84 (91%) of the acute infarcts. In the 8 remaining patients the infarcts were not detected by the automatic method due to their small size and low contrast differences on FLAIR images.

All patients had some level of WML. Volumes and relative volumes of both ischemic lesions and WML, as well as diffusivity values of the WML and NAWM are shown in Table 1. As expected, FA values were significantly lower and MD, Da and Dr values were significantly higher (*p*'s<0.001) in the WML compared with the NAWM.

### Age, gender, education and risk factors

#### Age

Age showed significant correlations with relative WML volume (r = 0.306, *p*<0.001) and WML diffusivity parameters: MD (r = 0.350, *p*<0.001), Da (r = 0.428, *p*<0.001) and Dr (r = 0.292, *p* = 0.001). No significant correlations were found between age and FA values in the WML and NAWM or with ILV.

#### Gender

Between genders comparison revealed no significant difference in age, years of education, cognitive scores, WML volume, ILV and diffusivity parameters of both WML and NAWM.

#### Education

Education showed positive correlations with global cognitive score (r = 0.432, *p*<0.001) and with all cognitive domains (rs>0.25 and *p*s<0.001).

No significant correlations were found between years of education and either WML volume or diffusivity parameters of WML and NAWM.

#### Risk Factors

The FSRP score did not correlate with any of the cognitive domains or ILV (for *p*<0.005). Significant correlations were observed between FSRP score and WML volume (r = 0.312, *p*<0.001) and all NAWM integrity parameters (rs>|0.28|, *ps*<0.001).

### Associations between white matter parameters and cognition

The correlations between tissue volumes (ischemic lesions and WML), WM tissue integrity (WML and NAWM) and cognitive domain scores at one year follow-up are shown in Table 2. The relative volume of ischemic lesions showed no correlation with any of the cognitive domains, while a larger relative volume of the WML was related to worse cognitive function in the global cognitive score, and specifically with memory, executive function and visual spatial domains. In addition, strong significant correlations were found between all NAWM diffusivity parameters and the global cognitive score. No correlations were found between tissue integrity within the WML and any of the cognitive domains.

**Table 2:** Correlation coefficients for the associations between ischemic lesions and white matter lesion volumes, white matter tissue integrity parameters and cognitive domain scores after one year.

	Ischemic lesions volume^a^^,^ b	WML volume^a^^,^ b	WML mean FA^b^	WML mean MD	WML mean Dr	WML mean Da	NAWM mean FA	NAWM mean MD	NAWM mean Dr	NAWM mean Da^b^
NeuroTrax total score (1 year)	−.15	−.32*	−.03	.01	.02	−.00	.27*	−.29*	−.31*	−.25*
Memory score (1 year)	−.13	−.26*	−.07	.00	.017	−.027	.23	−.29*	−.31*	−.26*
Executive function score (1 year)	−.20	−.28*	.01	.01	.01	.01	.26*	−.20	−.22	−.15
Visual spatial score (1 year)	−.23	−.40*	.03	−.05	−.04	−.05	.23	−.27*	−.28*	−.25*
Verbal function score (1 year)	−.02	−.17	−.01	−.05	−.05	−.06	.11	−.21	−.21	−.20
Attention score (1 year)	.00	−.17	−.05	.11	.11	.10	.25*	−.09	−.13	−.04

Abbreviations: NAWM, normal-appearing white matter; WML, white matter lesions; FA, fractional anisotropy; MD, mean diffusivity; Dr, radial diffusivity; Da, axial diffusivity.

a Data expressed as relative volume (percentage of intracranial volume).

b Logarithmic transformed data.

^*^ *p*<0.005.

### Associations between WML volume and WM tissue integrity

While WML volume was found to be highly correlated (rs>0.38, *p*s<0.005) with NAWM diffusivity parameters (MD, Dr and Da), no correlation was observed between WML volume and WML diffusivity parameters. Significant correlations (rs>0.26, *ps*<0.005) were found between WML diffusivity parameters (MD, Dr and Da) and NAWM diffusivity parameters (MD, Dr and Da), while no correlation was found for the FA values (Table 3).

**Table 3:** Associations between white matter lesion volume and white matter tissue integrity parameters.

	WML volume^a^^,^ b	WML mean FA^b^	WML mean MD	WML mean Dr	WML mean Da
WML volume^a^^,^ b	1	.02	.08	0.05	.13
NAWM, mean FA	−.21	.21	−.09	−.11	−.04
NAWM, mean MD	.39*	.04	.32*	.26*	.40*
NAWM, mean Dr	.38*	.02	.32*	.26*	.39*
NAWM, mean Da^b^	.39*	.05	.36*	.30*	.46*

Abbreviations: WML, white matter lesions; NAWM, normal-appearing white matter; FA, fractional anisotropy; MD, mean diffusivity;

Dr, radial diffusivity; Da, axial diffusivity;

a Data expressed as relative volume (percentage of intracranial volume).

b Logarithmic transformed data.

^*^ *p*<0.005.

### Association between ischemic lesions' location and cognition

ANOVA analysis revealed no significant difference in global cognitive score one year after the event between the four groups of patients (those with no observable new infarcts (n = 50, mean score 100 ± 13), those with cortical infarcts (n = 30, mean score 96±15), those with sub-cortical infarcts (n = 48, mean score 94±9) or those with subtentorial infarcts (n = 14, mean score 94±9)).

### Structural equation modeling results for the prediction of cognitive state

The previous analyses examined the predictive value of each single tissue parameter on cognitive state, while the following analysis integrates their effects using SEM.

Both models present a direct effect of education on cognitive state one year after the event (β = 0.328 and β = 0.343, *p*<0.001 for model 1 and 2 respectively). While age, in both models, has a significant indirect effect on the cognitive state through the mediating variables WML volume (β = 0.306, *p*<0.001) and NAWM integrity (β = 0.370, *p*<0.001), which in turn, significantly affect cognitive state (WML volume β = −0.231 and β = −0.203, *p*<0.05 and NAWM integrity β = −0.176 and β = −0.152, *ps*'<0.05, for models 1 and 2 respectively). ILV appears only in the first model and no significant direct effect on cognitive state was observed (β = 0.03).

Overall, the second model (Figure 2B), which did not include ILV, better fits the data (χ2(3) = 7.7, *p*>0.05, CFI = 0.9, GFI = 0.973, Adjusted GFI = 0.865, RMSEA = 0.11) compared to the first model (Figure 2A, χ2(3) = 45.89, *p*<0.001, CFI = 0.092, GFI = 0.873, AGFI = 0.388, RMSEA = 0.28).

These results indicate that ILV did not directly contribute to cognitive state one year after the event in our patient population.

### Multiple linear regression analysis for the prediction of cognitive state

Unadjusted regression analysis examined the contribution of each potential baseline variable to the global cognitive score one year after the stroke. Among all variables that were examined, age, education, FSRP score, ICV, WML volume and NAWM integrity were found to be associated with cognitive score and were entered into a multiple linear regression analysis as possible predictors. Results from multiple linear regression confirmed the relationships between WML volume (β = −0.25, SE = 1.88, *p*<0.005) and years of education (β = 0.35, SE = 0.25, *p*<0.001) with global cognitive score. These results supported the SEM results and explained ∼28% of the cognitive state variance (*p*<0.001). For more details see Table 4.

**Table 4:** Summary of unadjusted and adjusted regressions for predicting global cognitive score one year after stroke.

Unadjusted			
	β	SE	*p*
Age	−**0.198**	**0.108**	**0.018**
Gender	−0.121	2.08	0.153
Education	**0.409**	**0.259**	**<0.001**
Admission NIHSS	−0.087	0.366	0.331
Intracranial volume	**0.164**	**0.00**	**0.050**
WML volume	−**0.327**	**1.925**	**<0.001**
Ischemic lesions volume	−0.149	0.248	0.078
FSRP score	−**0.195**	**0.181**	**0.021**
NAWM integrity	−**0.272**	**1.0009**	**<0.001**
WML integrity	0.011	1.048	0.893

Adjusted (Model R^2^ = 0.281)			
	β	SE	*p*
Age	−0.052	0.141	0.629
Education	**0.347**	**0.252**	**<0.001**
FSRP score	0.19	0.240	0.866
Intracranial volume	0.116	0.000	0.131
WML volume	−**0.252**	**1.88**	**0.002**
NAWM integrity	−0.133	1.05	0.117

Abbreviations: NIHSS, National Institutes of Health Stroke Scale; WML, white matter lesions; FSRP, Framingham Stroke Risk Profile; NAWM, normal-appearing white matter.

### WML volume adjusted ANCOVA

While basic student t-test revealed a significant difference (*p* = 0.01) in cognitive performance between two groups defined by the presence or absence of ischemic lesions on DWI image (94.6±11.4 vs. 100.1±11.4, respectively), ANCOVA analysis controlling for WML volume eliminated this difference (F(139,1) = 2.0, p = 0.159).

These results suggest that only a minor effect of the ILV exist on cognition, in mild to moderate stroke patients.

## Discussion

Our results show the predominant role of preexisting WML and white matter integrity with no additional effect for ischemic lesions' volume and location, on cognitive state one year following first ever mild to moderate ischemic stroke or TIA. The relationship between WML load and cognitive state in post stroke patients has been demonstrated previously [19], [39]. However, the converging results of ANCOVA, regression analysis and SEM suggest that the occurrence of cognitive impairment in mild to moderate stroke or TIA survivors is related to preexisting WML rather than being directly affected by the stroke. The stroke itself, although possibly sharing with WML an etiology of underlying cerebrovascular disease, may represent an event that triggers and hastens the cognitive impairment process. Further studies are needed to investigate whether the same applies in cases of severe stroke.

In mild to moderate ischemic insults, the WML as well as NAWM integrity are important factors for patients' cognitive state [23], [45]. We have demonstrated that WML volume is negatively correlated with cognitive function one year after the event. This result provides additional support to the “disconnection hypothesis” in which WML disrupt pathways necessary for cognitive function [46].

We further demonstrate an association between the integrity of NAWM and cognitive scores, while no such correlations were observed for the integrity of the WML. These results are consistent only in part with a population based study of non-demented elderly [23] that demonstrated an association between microstructural integrity of both WML and NAWM and cognitive function. WML may be the result of different mechanisms (e.g. ischemia, inflammation, trauma, wallerian degeneration), and its integrity may reflect different degrees of damage. It may also be that once an insult to the WM is visible on conventional MRI (as WML), the microstructural integrity of this tissue reaches a ceiling effect, and thus, does not correlate with cognitive functioning of the brain. The integrity of the NAWM may be a better indicator for the “brain reserve capacity”, and may be a better predictor for cognitive state.

In addition, our results demonstrated an association between NAWM integrity and WML.

The evidence of abnormal WM integrity in the so-called NAWM surrounding WML was reported previously by Maillard *et al.* [24] and supports the notion that WMLs fail to capture the full extent of WM injury. To date, few studies have investigated the relationship between changes in the NAWM tissue integrity parameters at baseline and the development of WML volume [16], [47]. These longitudinal studies provide evidence that the WM injury process is continuous in time. The risk of a WM voxel converting into a WM hyperintense lesion at follow-up increases continuously with a continuous decrease in baseline WM integrity [16], [48]. Maillard *et al.* [48] also suggest that DTI and FLAIR provide complementary information for capturing the time course of WM degeneration, and therefore have potential as biologic markers of vascular brain disease. Our results are consistent with previous studies [49], [50] and may strengthen the hypothesis that impaired NAWM tissue integrity may in turn result in WML, and further suggest that detecting abnormalities in NAWM integrity may identify which subjects are vulnerable to develop cognitive dysfunction via the *NAWM tissue integrity → WML → cognitive decline* pathway, regardless of the occurrence of an ischemic event. Additional longitudinal studies are required in order to confirm this hypothesis.

The impact of vascular risk factors on post stroke cognitive impairment is debatable. The Sydney stroke study [9] showed that cerebrovascular risk factors are not independent predictors of post stroke cognitive impairment, while other studies demonstrated indirect evidence for high blood pressure, diabetes and IHD as risk factors for post stroke cognitive impairment [42], [51]. In the present study, only non-significant associations (for p<0.005) were found between FSRP score and any of the cognitive domains. WML are often associated with vascular risk [12], [18]. Age, hypertension, smoking, diabetes mellitus, cholesterol and history of vascular disease were all shown to be associated with lesions in WM [12]. In this study, as in previous research, significant associations were found between FSRP score and WML volume and all NAWM integrity parameters.

Worthy of comment is the lack of age effect on cognitive score (for *p*<0.005) and the absence of a direct relation between age and cognitive state in SEM models. As previously reported [52], [53], age has a direct effect on cognitive function. However, since the NeuroTrax computerized cognitive testing scores are already normalized to an age-matched healthy population, we did not expect to see significant correlations between age and the different cognitive scores. Additionally, in order to avoid over-adjustment and increase of net bias, age was not entered directly to the SEM models [54].

Finally, the multiple regression analysis demonstrated that WML volume and years of education have predictive value for global cognitive score one year after stroke. The assumptions for multiple regression analysis include suitable specification of the model, linear relationships and the same level or relation through the range of independent variables. SEM, on the other hand, permits complicated variable relationships to be expressed through hierarchical or non-hierarchical, recursive or non-recursive structural equations, to present a more complete picture of the entire model. In SEM, it is necessary to begin with theory guided hypotheses [31]. Here, the main hypotheses, and hence the two driven models, rely on the debatable role of ischemic lesions on cognitive state following the event. Furthermore, SEM models should be parsimonious, containing only the relevant relationships which are more likely to be generalizable [31]. SEM results indicate that new ischemic lesions play a minor role, if any, in cognitive outcome, compared to the impact of the preexisting WML volume and NAWM integrity, in mild to moderate stroke patients.

The strengths of this study include the relatively large, single center, prospective, first ever stroke or TIA cohort; the identical 3T MRI protocol used for all patients; and the extensive assessment of cognitive performance. In addition, MRI analyses of WML, ischemic lesions and NAWM integrity were reported using validated semi automated quantitative assessments. This method is replicable and takes into account the presence of preexisting disease that may confound volume measurements [34].

Several limitations to this study should be considered. First, DTI parameters in WML and NAWM are reported as global mean thus might be insensitive for the detection of changes occurring at specific fiber tracts. Second, although this study, as is common in the literature, accounts for the overall WML load, it is also possible that WML location plays an additional role. Third, our study includes only mild to moderate stroke patients who are expected to be able to perform cognitive tests and be available for follow-up. Fourth, our patients were highly educated and the results may be different for less educated individuals and those whose risk factors are less well controlled. Fifth, in this study we acknowledge the lack of “normal” controls. However, although no direct comparison with a control group was made, the NeuroTrax computerized cognitive testing scores are already normalized to an age-matched normal population [35]. Sixth, only patients who were free from cognitive decline before the event were included in this study (as determined by the IQCODE, Informant Questionnaire on Cognitive Decline in the Elderly). Yet, the cognitive state of our patients before the ischemic event was not systematically evaluated; hence, we used the term cognitive state at one year follow-up rather than cognitive decline. Finally, this study lacks imaging data at one year (when the cognitive assessment was performed) and thus, confounders that may have developed during the one year time gap between MR data acquisition and the cognitive tests, factors like recurrent strokes, emergence of new silent infarcts, progression of small vessel disease, etc., could not be evaluated and taken into account. These confounders, if they occurred, may affect the cognitive state after one year.

## Summary

Our findings show that mild to moderate stroke patients with WML are more vulnerable to cognitive impairments regardless of their new ischemic lesions' volume and location. Thus, these patients can serve as a target group for studies on cognitive rehabilitation and neuro-protective therapies which may, in turn, slow their cognitive deterioration.
